# Supplementary material for: Culinary Comfort: Understanding the Connection between Food and Well-Being
Source: Nutrients. 2024 Jun 14;16(12):1865. doi: 10.3390/nu16121865 (PMC11206277; doi:10.3390/nu16121865)
Supplement: Supplementary file 1 [file nutrients-16-01865-s001.zip › nutrients-3050803-supplementary.pdf]

Questionnaire
Culinary Comfort: Understanding the Connection between Food and Well-being

Section S1: Demographic

Profile Gender:

1. Male_________
2. Female___________

Area (belonged to):

1. Urban_________
2. Semi-Urban__________
3. Rural_________

Age (Years)

1. 18-29___________
2. 30-41___________
3. 42-54___________
4. 55 & above________

Education

1. Informal
2. Primary
3. Secondary
4. Intermediate
5. Graduation
6. Post-Graduation

Income

1. Rs.25000/-50000/-
2. Rs. 50001/-100,000/-
3. Rs. 100,001/-150,000/-
4. Rs. 150,001/- & above

Section S2: Food Choice and Well-Being

Important Factors influence/shaping choices towards contemporary cuisine		Not Important at All (1)	A Little Important (2)	Moderately Important (3)	Very Important (4)
1	Ready to eat/prepared				
2	Contains no additives				
3	Low in Calories				
4	Taste good				
5	Contains natural ingredients				
6	Is not expensive				
7	Is low in fat				
8	Is familiar to me				
9	Is high in fiber and roughage				
10	Is nutritious				
11	Is easily available in shops and supermarkets				
12	Is value good for money				
13	Cheers me up				
14	Smells nice				
15	Can be cooked very easily/simply				
16	Helps me to cope with stress				
17	Helps me control my weight				
18	Has a pleasant texture				
19	Is packaged in an environmental friendly way				
20	Comes from countries I approve of politically				
21	Is like the food I ate when I was a child				
22	Contains lots of vitamins and minerals				
23	Contains no artificial ingredients				
24	Keep me awake and alert				
25	Looks nice				
26	Helps me relaxed				
27	Is high in protein				
28	Takes no time to prepare				
29	Keeps me healthy				
30	Is good for my skin/hair/nails etc				
31	Makes me feel good				
32	Has country of origin clearly marked				
33	Is what I usually eat				
34	Helps me to cope with life				
35	Can be bought in shops where I live or work				

Section S3: Kitchen Practices

**Kitchen Values**		**Never (1)**	**Seldom (2)**	**Sometime (3)**	**Often (4)**
1	Like family recipes				
2	Like family food ingredients				
3	Prefer homemade food				
4	Like home food flavors				
5	Remember grandmother food taste				
6	Prefer grandmother recipes				
7	Prefer traditional foods				
8	Ask traditional food on special occasions				
9	Recall/share food related memories during dinning				
10	Get enjoyment				
11	Enhance health				
12	Cooking activities enhance home livelihood/Gorgeousness				
13	Creates bonds(strengthen relationships by encouraging a sense of trust, belongingness, and closeness)				
14	Improves quality of life (save money, provide energy)				
15	Creates a space for conversation				
16	Promotes healthier eating habits				
17	Supports self-care (raise self-esteem)				
18	Support family care (confidence in the development of new skill and accomplishing a task with a reward)				
19	Facilitates a healthier life style				
20	Improve concentration and reduce stress				

Section S4: Family Ties and Well-Being

Social Relationships/Family Ties		Never	Seldom	Sometimes	Often
1	How often you take breakfast with your family?				
2	How often you take lunch with your family?				
3	How often you take dinner with your family?				
4	How often family members complements your attempts to dine with them?				
5	How often family members complements your attempts for healthy diet?				
6	How often family members make you feel happy eating with them?				
7	How often family members make you feel guilty not eating with them?				
8	How often family members appreciate your food choice?				
9	How often family members encourage your food choice?				
10	How often family members talk about food and nutrition with you?				
11	How often you bring fruits for family members to dine?				
12	How often you bring vegetables for family members to cook?				

13	How often you participate in cooking?				
14	How often family members share their feelings while dine?				
15	How often family members share their worries while dine?				
16	How often family members and you share memories while dine?				
17	Is cooking a way you show and receive care?				
18	Do you feel stressed when you eat a home-cooked meal?				
19	I feel satisfied when I eat a home-cooked meal native to my culture?				
20	In my life, dine together is a gift way to celebrate method of showing care?				
21	In my life, dine together is a way to feed myself and others?				
22	Dine together is a hassle way to relax Expression of creativity?				
23	Dine together is a way to bring people together?				
24	Dine together is a waste of time?				
25	Through dinning and cooking, I feel connected with my family?				
26	Cooking and dinning traditions are an important part of my family?				

27	Cooking and dinning traditions are an important part of my culture?				
28	Has cooking and dinning together affected your family relationships?				
29	Has cooking and dinning affected your cultural identity development?				
30	Dine together provides feelings/ sense of more belongingness?				
31	Dine together is a place to get support, love and affection?				
32	Cooking and dinning foster positive outlooks about self and strong family ties?				
33	Dinning and cooking improve quality of relationships?				
34	Dine together uncovers the needs to shape/asses individuals' point of views?				
35	Enhance well-being?				
